# Surveillance for Antimicrobial Drug Resistance in Under-Resourced Countries

**DOI:** 10.3201/eid2003.121157

**Published:** 2014-03

**Authors:** Guy Vernet, Catherine Mary, Dany M. Altmann, Ogobara Doumbo, Susan Morpeth, Zulfiqar A. Bhutta, Keith P. Klugman

**Affiliations:** Fondation Mérieux, Lyon, France (G. Vernet);; Avicenne, Lyon (C. Mary);; The Wellcome Trust and Imperial College, London, UK (D.M. Altmann);; Malaria Research and Training Center, Bamako, Mali (O. Doumbo);; Kenya Medical Research Institute–Wellcome Trust Research Programme, Kilifi, Kenya (S. Morpeth);; University of Oxford, Oxford, UK (S. Morpeth);; The Aga Khan University, Karachi, Pakistan (Z.A. Bhutta);; Emory University, Atlanta, Georgia, USA (K.P. Klugman);; University of the Witwatersrand, Johannesburg, South Africa (K.P. Klugman)

**Keywords:** antimicrobial resistance, surveillance, under-resourced countries, diagnostics

## Abstract

Antimicrobial drug resistance is usually not monitored in under-resourced countries because they lack surveillance networks, laboratory capacity, and appropriate diagnostics. This accelerating problem accounts for substantial number of excess deaths, especially among infants. Infections particularly affected by antimicrobial drug resistance include tuberculosis, malaria, severe acute respiratory infections, and sepsis caused by gram-negative bacteria. Nonetheless, mapping antimicrobial drug resistance is feasible in under-resourced countries, and lessons can be learned from previous successful efforts. Specimen shipping conditions, data standardization, absence of contamination, and adequate diagnostics must be ensured. As a first step toward solving this problem, we propose that a road map be created at the international level to strengthen antimicrobial resistance surveillance in under-resourced countries. This effort should include a research agenda; a map of existing networks and recommendations to unite them; and a communication plan for national, regional, and international organizations and funding agencies.

## Abstract 2

TOC summary: New programs can be improved by drawing on lessons from previous successful efforts.

Antimicrobial drug resistance has become such a global concern that it was the focus of the 2011 World Health Day sponsored by the World Health Organization (WHO). Although antimicrobial drug resistance is well mapped and tightly monitored in some well-resourced countries, such processes do not exist in under-resourced countries. An increasing body of evidence reveals accelerating rates of antimicrobial drug resistance in these countries. Resistance may arise in the absence of any surveillance and threatens the achievement of the Millenium Goals for Development in terms of reduction of maternal and infant deaths (www.un.org/millenniumgoals/). The problem is even more pressing because, in a globalized world, microorganisms and their resistance genes travel faster and farther than ever before, and the pipeline of new drugs is faltering.

Mapping antimicrobial drug resistance in under-resourced countries is urgently needed so that measures can be set up to curb it. Such mapping must rely on efficient surveillance networks, endowed with adequate laboratory capacity, and take into account up-to-date diagnostic techniques. The way forward is to assess the effects of resistance, its clinical effects, and increase in deaths, with the ultimate objective of providing achievable guidelines for surveillance and control.

In this article, we report examples of successful surveillance networks in under-resourced countries and address the framework upon which to deploy reliable and sustainable networks on antimicrobial drug resistance surveillance. This initiative was begun during the Expert Meeting on Diagnosis and Detection of Antimicrobial Resistance in Developing Countries, convened at the Fondation Mérieux Conference Centre “les Pensières,” Veyrier-du-Lac, France, October 26–28, 2011.

## What Are the Main Threats?

### Tuberculosis

Resistance *of Mycobacterium tuberculosis* to antimycobacterial drugs is a global concern (Figure). In 2010, an estimated 650,000 cases of multidrug-resistant tuberculosis (MDR TB) (i.e., infections with strains resistant to, at minimum, rifampin and isoniazid) occurred worldwide, (*1*). An estimated 10% of cases were extensively drug resistant (XDR) (i.e., MDR strains that are also resistant to second-line drugs). Almost no surveillance system is in place and no data exist on TB resistance in sub-Saharan Africa (apart from South Africa) and Asia.

**Figure:**
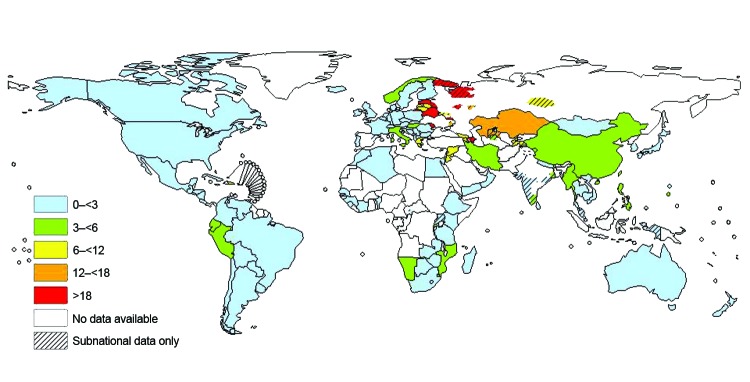
Proportion of multidrug resistance among strains causing new tuberculosis cases from latest available world data, 1994–2010.  Dotted lines on maps represent approximate borders for which there may not yet be full agreement. (Copyright by the World Health Organization; 2011. All rights reserved.)

### Malaria

*Plasmodium falciparum* strains resistant to chloroquine, fansidar, and mefloquine are widespread. Interventions using artemisinin and insecticide-treated bed nets have led to a drop of 40% of malaria cases since 2004 according to WHO. An estimated 750,000 lives were saved in Africa alone (*2*). Those efforts are potentially hindered by the emergence of resistance to artemisinin, which was first reported in 2008 at the Thailand–Cambodia border and subsequently reported in neighboring countries, although not in Africa so far (*3*,*4*). Resistance mechanisms to artemisinin are poorly understood, although mutations in some parasite genes have been partially correlated with resistance (*5*,*6*).

### Severe Acute Respiratory Infections 

Severe acute respiratory infections (SARIs) kill an estimated 1.4 million children <5 years of age every year (*7*). The emergence of resistance to neuraminidase inhibitors would potentially have dramatic consequences because they are the first-line response to pandemics caused by highly virulent influenza virus. Resistance of *Streptococcus pneumoniae* to antimicrobial drugs is also a concern. The extent of outpatient penicillin usage correlates with level of resistance (*8*). A prospective surveillance study of 2,184 patients hospitalized with pneumococcal pneumonia in 11 Asian countries in 2008–2009 found that high-level penicillin resistance was rare, that resistance to erythromycin was highly prevalent (72.7%), and that MDR was observed for 59.3% of *S. pneumoniae* isolates (*9*). Of 20,100 cases of invasive pneumococcal diseases identified in South Africa during 2003–2008, a total of 3,708 (18%) were caused by isolates resistant to at least 3 antimicrobial drugs (*10*).

### Gram-negative Bacteria Infections

Gram-negative bacteria resistant to β-lactams are spreading worldwide. CTX-M-15, a heterogeneous and mobile resistance gene first described in 2001 in India, has since been reported all over the world and is transmissible between different species of *Enterobacteriaceae* (*11*). New Delhi metallo-β-lactamase-1 (NDM-1) is a gene that confers resistance to all β-lactams, including carbapenems, which are the only alternative for treating severe infections such as neonatal sepsis caused by MDR strains. First identified in 2008, it is now widespread in *Escherichia coli* and *Klebsiella pneumoniae* isolates from the Indian subcontinent and is found in many countries (*12*–*18*). Spread of gram-negative resistant bacteria from the hospital to the environment by direct person-to-person contact or through nonsanitized water is a concern in under-resourced countries (*19*). The worldwide increase of the number of travelers, some of whom have diarrhea, is a major cause of the spread of resistance. A study conducted in Barcelona, Spain, showed that nalidixic acid resistance in enterotoxigenic or enteroaggregative *E. coli* strains isolated from patients returning from India increased from 6% during 1994–1997 to 64% during 2001–2004. Sixty-five percent of strains isolated from patients who had traveled to India were resistant to quinolones (*20*).

### Methicillin-Resistant *Staphylococcus aureus* Infections

Methicillin-resistant *Staphylococcus aureus* (MRSA) infections have become widespread even in under-resourced countries (*21*,*22*). Pakistan and India have reported MRSA percentages of 42%–54.9%, with an increasing trend (*23*). 

The above sections describing antimicrobial drug resistance in various diseases is not exhaustive. Careful attention must be given to the potential spread of antimicrobial drug resistance in the drugs used to treat highly prevalent infectious diseases such as typhoid, meningitis, HIV, and hepatitis in under-resourced countries.

## Who Are the Most Vulnerable Populations?

Antimicrobial drug resistance accounts for excess deaths in infants and childbearing women because of poor intrapartum and postnatal infection–control practices (*24*,*25*). In 2005, infections in hospital-born babies were estimated to account for 4% to 56% of all deaths in the neonatal period in some under-resourced countries (*24*). *K. pneumoniae*, *E. coli*, *Pseudomonas* spp., *Acinetobacter* spp., and *S. aureus* were the most frequent causative pathogens of neonatal sepsis; 70% of these isolates would not be eliminated by an empiric regimen of ampicillin and gentamicin. Many infections might be untreatable in resource-constrained environments. Fifty-one percent of *Klebsiella* spp. were extended-spectrum β-lactamase (ESBL) producers, 38% of *S. aureus* strains were methicillin-resistant, and 64% were resistant to co-trimoxazole (*24*). Preliminary data from Kilifi District Hospital (Kenya) also show alarming rates of ESBL positivity: 180 (39%) of 459 *Enterobacteriaceae* clinical isolates from child and adult patients (including 115 isolates of *K. pneumoniae*) collected from August 2010 to August 2012 were ESBL positive (S. Morpeth, unpub. data).

The Division of Women and Child Health at the Aga Khan University Medical College in Karachi, Pakistan, has proposed a model for monitoring the development of neonatal infections and outcomes in southern Asia, on the basis of a cohort of 69,450 births. Resistance rates are constantly increasing. Antimicrobial drug resistance is estimated to result in an additional 96,000 (≈26%, range 16%–37%) deaths each year from neonatal sepsis in southern Asia, highlighting the toll that children pay for drug resistance (Z. Bhutta, unpub. data).

## Promises and Limits of Molecular Diagnostics

The diagnosis of resistance to antimicrobial drugs has so far relied on culture techniques performed in reference centers; these procedures have a long turnaround-time, are technically demanding, and are sometime dangerous. *M. tuberculosis* is difficult to grow, and *Mycobacterium leprae*, *Treponema pallidum,* hepatitis B virus, *Chlamydia pneumoniae*, *Chlamydia trachomatis,* and HIV are impossible to grow. Molecular diagnostic tools offer new promise.

The recent implementation of molecular tools for the diagnosis of *M. tuberculosis* infection and resistance in under-resourced countries illustrates the importance of integrating these tools with traditional diagnostic methods. Mutations within the *M. tuberculosis* genome are associated with resistance. Two PCR-based molecular diagnostic tests were recently introduced with the endorsement of WHO and the financial support of international organizations. GenoType MTB-DRplus (Hain Lifescience, Nehren, Germany) identifies the infectious agent and detects resistance to rifampin and isoniazid in only 2 days with sensitivity and specificity in smear-negative sputum of 94.3% and 96.0%, respectively, compared with drug susceptibility tests (DST) (*26*). A recently released version has sensitivities for the detection of resistance to second-line drugs ranging from 57% to 100% (*27*). Xpert MTB/RIF (CEPHEID, Sunnyvale, CA, USA) diagnoses infection and detects resistance to rifampin with sensitivity and specificity of, respectively, 99.1% and 100%, in 2 hours and in a closed cartridge by using a fully integrated analyzer (*28*).

An immunochromatographic qualitative assay for identifying MRSA, which detects penicillin-binding protein 2a from *S. aureus* isolates (not on specimens), is sold by Alere Inc. (Waltham, MA, USA). CEPHEID produces 2 GeneXpert cartridges to detect MRSA and vancomycin-resistant enterococci on specimens and is currently developing a cartridge for simultaneous detection of 3 major β-lactamase genes (KPC 1–11, NDM 1–6, and VIM 1–32) in rectal swab specimens. Resistance to antiretroviral drugs is usually determined by gene sequencing. None of these assays are extensively used in under-resourced countries.

However, as promising as these diagnostics are, their use would help identify resistance for a limited number of microorganisms. The multiplicity of resistance genes, low inoculum size, variations in permeability to antibacterial drugs, and the activity of efflux pumps are factors influencing antibacterial drug sensitivity. Development of new assays will require research efforts. A novel approach, based on RNA detection of a set of bacterial transcripts after a brief period of antibacterial pulse therapy, may enable rapid differentiation of susceptible and resistant organisms directly from specimens (*29*). Nonetheless, molecular diagnostic tests should not replace culture-based DST in central reference laboratories that are responsible for updating treatment algorithms and identifying new resistance mechanisms.

## Mapping Antimicrobial Drug Resistance 

### Resistance Surveys in Africa and Southeast Asia

A surveillance network, coordinated by the Malaria Research Training Centre (University of Bamako, Bamako, Mali), has taken advantage of rapid molecular-based tests that have been adapted for field use. It relies on health care workers, who have limited clinical training. After a short training course, they are able to collect blood samples from finger pricks and spot them onto filter paper strips that are sent to regional sites that detect PfCRT 76T, the key mutation causing chloroquine resistance. Data are centralized to establish a comprehensive map of resistance. Overall, results showed high prevalence rates (84.5%; range 60.9%–95.1%) across the sites (*30*). Another survey in the district of Kidal identified the mutation in 80% of cases and showed that no isolates carried the dhfr/dhps quintuple mutant (a genetic determinant of pyrimethamine resistance) (*31*). On the basis of these results, chloroquine was replaced by sulfadoxine-pyrimethamine for malaria treatment. Key factors that contributed to the success of this health information system included molecular methods and the reliability of the data-sharing system. Internet access through satellite connection, which became possible a few years ago, will allow more timely data centralization.

A project in 8 West African countries, coordinated by Institut Pasteur and the International Union Against Tuberculosis and Lung Diseases and funded by the Organization of the Petroleum Exporting Countries Fund for International Development, plans to implement GenoType MTBDRplus (Hain Lifescience) on samples positive for *M. tuberculosis* by miscroscopy and Xpert MTB/RIF (CEPHEID) on samples from persons who experienced treatment relapse or failure. These methods will be used in reference centers, together with methods already in use. Another successful example is the South African National TB Drug Resistance Survey (www.mrc.ac.za/operationaltb/DRSUpdate.pdf), which started in June 2012 and plans to enroll 170,000 patients with suspected TB; culture DST will be performed by a sentinel reference laboratory. The survey is supported by several national and international funding agencies. Similarly, the Asian Network for Surveillance of Resistant Pathogens implemented an extensive survey of antimicrobial drug resistance in isolates causing pneumococcal infections in 11 Southeast Asian countries (*9*).

### Role of Health and Demographic Surveillance Sites (HDSS)

Grundmann et al. have published an inventory of preexisting regional surveillance programs in the 6 WHO regions (*32*). The authors suggest that HDSS could serve as focal points for training and dissemination of laboratory and surveillance competencies. HDSS have been established in 39 countries in Asia, Africa, and Oceania as part of the International Network for the Demographic Evaluation of Populations and Their Health in Under-Resourced Countries initiative (INDEPTH; www.indepth-network.org/). HDSS monitor >2 million persons at the household level on a regular basis, have good management capacities, and, as such, create an opportunity for monitoring antimicrobial drug use at the consumer level. A study among children <5 years of age in Kilombero (Tanzania) shows that mapping the patterns of antimicrobial drug use and monitoring resistance is possible based on HDSS, even in a region with only 1 doctor for a population of ≈1 million persons (*33*). In this region, maps, which included houses, hospitals, drug shops, villages, health centers, and the level of antimicrobial drug use revealed that the poorer the family, the less access they had to antimicrobial drugs for their children. On the basis of this map, antimalarial drugs and antibacterial drugs were distributed to the population.

There is considerable potential for use of sentinel population-based studies with monitoring of febrile illnesses (*34*) or routine or diarrheal diseases. Blood cultures and stool specimens can be used to evaluate the prevalence of antimicrobial drug resistance in urban and rural settings (*35*).

### Using Existing Databases 

The above-mentioned survey conducted in Karachi emphasizes the need to systematically assess available information from databases such as a hospital’s mainframe admissions database, the neonatal intensive care unit admission database, and the microbiology laboratory database to evaluate potential links between antimicrobial drug resistance and major infectious diseases. However, such modeling exercises need to be complemented with prospective surveillance studies.

When measuring antimicrobial drug resistance in under-resourced countries, researchers should consider the epidemiology of the infections and what the denominator is for the surveillance. Too often, published antimicrobial drug resistance data come from hospitals that only wealthy patients can afford to attend, which biases the data.

## Addressing Basic Problems

###  Insufficient Laboratory Capacity 

Lessons learned from antimicrobial drug resistance surveillance highlight the need to endow surveillance networks in under-resourced countries with laboratory capacity in a sustainable manner—infrastructure and human resources are required to obtain reliable data that can inform both clinicians and policy makers. Common problems should be addressed first: the death of bacteria during shipping, lack of standardization and guidelines for preparing culture media, high rates of culture contamination, and the lack of availability of diagnostic tests matching the most common infectious agents in circulation in a given area. A survey within the Network for Surveillance of Pneumococcal Disease in the East African Region shows, for example, that an average of 17% of isolates died during shipping (4%–7% from Kenya and nearby Tanzania and 20%–60% from sites further away) and that a frequent cause of this wastage was the shortage of dry ice to keep the isolates frozen (*36*). Other common problems include the lack of harmonization of methods of culture used to grow bacteria, frequent unavailability of required laboratory consumables due to lack of resources and supply-chain problems, and inaccurate training and poor motivation of laboratory technicians and clinicians.

Training, feedback, and the introduction of a few simple hygienic measures implemented over the past 3 years in the pediatric service at Kilifi District Hospital have resulted in a reduction of the rates of contamination in blood culture samples from children from 19% in January 2009 to 5% in July 2011 (S. Morpeth, unpub. data). Good coordination between clinical and laboratory staff was crucial for this achievement, but the backing of the hospital senior clinical management staff was also necessary. However, the effort required to achieve similar results in standard district hospitals will obviously be more intense than it was at Kilifi District Hospital, which is part of a major research program. Clinical demand and political will are critical for the development of adequate laboratory services.

Existing infrastructures in under-resourced countries encompass ingredients for good surveillance of antimicrobial drug resistance, but the bottleneck is laboratory capacity. This capacity needs to be built into all levels of the health care system. Planning should take into account the following: tools to be used for diagnosis, sustainable funding, sharing of standard operating procedures, data management, a central database, and resource centers. In addition, logistical and human issues should be considered: for example, common procurement strategies, training of technicians, communication to raise awareness of antimicrobial resistance, feedback of results to local clinicians and central policy makers, and identification of the leaders who will support the development of surveillance networks.

### Standardization of Resistance Data

Standardization is critical for comparison and globalization of surveillance. The Clinical and Laboratory Standards Institute in the United States and the European Union Commission do not agree on the values of MICs or disk diffusion zone size break points for antibacterial drugs, and the introduction of molecular techniques may further complicate comparisons. Setting up a resistance index that would make the data comparable across different regions would be a valuable research objective.

## Building on Successful Experiences

### European Antimicrobial Resistance Surveillance Scheme (EARSS)

Drawing on lessons learned from the development of EARSS, a surveillance system that monitors antimicrobial drug resistance in Europe and other networks, we strongly recommend that countries start with good quality data from a limited number of sites. Similarities between the situation in Europe when EARSS started its surveillance activities and the situation in Africa can be pointed out. EARSS has been gradually scaled up from a starting point in 1998 when only 2 bacteria were monitored in 78 laboratories in 7 countries. In 2011, EARSS encompassed 977 laboratories and 1,577 hospitals in 33 countries covering a population of >700 million citizens (*37*). This experience could guide the development of surveillance in under-resourced countries.

### Using Existing Networks 

Surveillance networks developed for specific purposes could serve as the basis for monitoring other infectious agents and their resistance to antimicrobial drugs. For example, initiatives are ongoing within the Organisation de Coordination pour la Lutte contre les Endémies en Afrique Centrale to foster the awareness of antimicrobial drug resistance in Africa (B. Gicquel, unpub. data). Similarly, blood culture facilities installed as part of malaria vaccine trials provide a background infrastructure that could be built on (*32*).

## Perspectives and Recommendations

The Table shows recommendations from the group that met at the Expert Meeting on Diagnosis and Detection of Antimicrobial Resistance in Under-Resourced Countries. A roadmap for the development of effective surveillance networks, endowed with good laboratory capacities, is urgently needed. Surveillance should also be integrated with other public health measures aimed at curbing the spread of pathogens, such as vaccination campaigns. Conjugate vaccines, for example, have been shown to reduce the antibacterial drug resistance of *S. pneumoniae* strains (*38*). Surveillance should thus be part of a global and coordinated strategy for control of antimicrobial drug resistance in under-resourced countries. The roadmap should follow a gradual process, starting with a limited number of pathogens, because attempting to address everything everywhere could kill the project. The meeting participants agreed on the necessity of establishing the roadmap with 3 key items: a research agenda, a map of existing initiatives and networks, and a communication plan aimed at international organizations and funding bodies. 

**Table:** Recommendations for efficient antimicrobial drug–resistance surveillance in under-resourced countries

Recommendation
Laboratory improvement
Address both patient management and surveillance needs
Build sustainable capacity (infrastructure, equipment, human resources)
Provide good coordination between clinics and laboratory
Standardize procedures
Identify appropriate diagnostic tests for antimicrobial drug resistance (e.g., molecular tests for uncultivable or slow-growing bacteria or for organisms in which resistance is linked to a single gene
Logistical needs
Avoid shortage of reagents; address both resources and supply chain Ensure appropriate specimen collection and transport to the laboratory
Political will
Backed by hospital management
Endorsed by policy makers
Standardized antimicrobial drug resistance results: resistance index
Leverage successful experiences
Integrate drug resistance surveillance to other public health measures aimed at curbing the spread of pathogens
Start small; increase gradually
Take advantage of existing networks targeting specific diseases (HIV, malaria, tuberculosis)

### Research Agenda

The research agenda should focus on developing appropriate technology for antimicrobial drug resistance detection, leveraging novel technologies that have emerged recently. The group will review these technologies and build a proposal for funding agencies. 

### Mapping Existing Initiatives

Mapping existing initiatives will be a key component of the roadmap. Recently, several such initiatives have been launched. In 2011, WHO emphasized the need to strengthen efforts recommended in its 2001 Global Strategy for Containment of Antimicrobial Resistance, for example through the strategic action plan on antimicrobial drug resistance of the WHO European region. The WHO Patient Safety Program published in 2012 The Evolving Threat of Antimicrobial Resistance: Options for Action (www.who.int/patientsafety/implementation/amr/publication/en/), which lists some networks involved in antimicrobial drug resistance surveillance activities, including those in Africa, Southeast Asia, and Latin America. In 1911, the European Union (EU) proposed an Action Plan against the rising threat of antimicrobial drug resistance. Several EU initiatives should support activities in under-resourced countries: the Joint Programming Initiative on Antimicrobial Resistance (http://www.jpiamr.eu/) gathered 17 EU countries and proposes to set 10 million €/year for 5 years for research; the Innovative Medicine Initiative, a public–private partnership between EU and the pharmaceutical industry association; the European Federation of Pharmaceutical Industries and Associations, which edits regular calls for proposals within its program Combating Antibiotic Resistance: New Drugs for Bad Bugs,the next EU program for research and innovation (Horizon 2020); and the European and Under-resourced Countries Clinical Trials Partnership. EU and the United States support the Transatlantic Task Force on Antimicrobial Resistance, which recently reviewed its recommendations to keep antimicrobial agents effective (www.ecdc.europa.eu/en/activities/diseaseprogrammes/TATFAR/ Documents/210911_TATFAR_Report.pdf). Most of these initiatives are targeting the well-resourced world. There is an urgent need to advocate for consideration of specificities and constraints of preventing antimicrobial drug resistance, especially surveillance, in low- and middle-income countries and to implement existing global plans, with substantial and sustainable funding provided to these countries. 

### Detailed Communication Plan

Advocacy for surveillance for antimicrobial drug resistance, based on a detailed communication plan, should also target health authorities in the concerned regions. The Health Ministers of WHO South-East Region member states agreed in 2012 on 18 recommendations at national and regional levels for preventing and containing antimicrobial drug resistance, including increased capacity for surveillance (http://209.61.208.233/LinkFiles/WHD-11_antimicrobial drug resistance_Jaipur_declaration_.pdf). This call for action should be reproduced in other WHO regions, especially in the region for Africa.

To build this 3-item roadmap, the proposed initiative should join efforts with programs like the Global Antibiotic Resistance Partnership, which develops actionable policy proposals on antibiotic resistance for low- and middle-income countries through a network with members in India, Kenya, South Africa, Vietnam, Mozambique, and Tanzania (www.cddep.org/projects/global_antibiotic_resistance_partnership); GABRIEL (Global Approach for Biological Research on Infectious Epidemics in Low-Income Countries), coordinated by Fondation Mérieux, with laboratories in 17 under-resourced countries; or the International Network of Pasteur Institutes, found in 32 countries.
